# Enhancing perinatal health patient information through ChatGPT – An accuracy study

**DOI:** 10.1016/j.pecinn.2025.100381

**Published:** 2025-02-10

**Authors:** P.L.M. de Vries, D. Baud, S. Baggio, M. Ceulemans, G. Favre, E. Gerbier, H. Legardeur, E. Maisonneuve, C. Pena-Reyes, L. Pomar, U. Winterfeld, A. Panchaud

**Affiliations:** a Department of Gynecology and Obstetrics, Lausanne University Hospital and University of Lausanne, Lausanne, Switzerland; b Institute of Primary Health Care (BIHAM), University of Bern, Bern, Switzerland; c Laboratory of Population Health (#PopHealthLab), University of Fribourg, Fribourg, Switzerland; d Clinical Pharmacology and Pharmacotherapy, Department of Pharmaceutical and Pharmacological Sciences, KU Leuven, 3000 Leuven, Belgium; e L-C&Y, Child and Youth Institute, KU Leuven, 3000 Leuven, Belgium; f Department for Health Evidence, Radboud University Medical Center, 6500 HB Nijmegen, the Netherlands; g Graduate School for Health Sciences (GHS), University of Bern, Bern, Switzerland; h Institute for Information and Communication Technologies (IICT), School of Engineering and Management Vaud (HEIG-VD), HES-SO University of Applied Sciences and Arts Western, Switzerland; i Computational Intelligence for Computational Biology (CI4CB), Swiss Institute of Bioinformatics (SIB), Lausanne, Switzerland; j School of Health Sciences (HESAV), University of Applied Sciences and Arts Western Switzerland, Lausanne, Switzerland; k Swiss Teratogen Information Service and Clinical Pharmacology Service, Centre Hospitalier Universitaire Vaudois (CHUV) and University of Lausanne, Lausanne, Switzerland; l Service of Pharmacy, Lausanne University Hospital and University of Lausanne, Lausanne, Switzerland

**Keywords:** Perinatal health information, Artificial intelligence, ChatGPT, Patient information, Pregnancy, Nutrition, Warning signs

## Abstract

**Objectives:**

To evaluate ChatGPT's accuracy as information source for women and maternity-care workers on “nutrition” and “red flags” in pregnancy.

**Methods:**

Accuracy of ChatGPT-generated recommendations was assessed by a 5-point Likert scale by eight raters for ten indicators per topic in four languages (French, English, German and Dutch). Accuracy and interrater agreement were calculated per topic and language.

**Results:**

For both topics, median accuracy scores of ChatGPT-generated recommendations were excellent (5.0; IQR 4–5) independently of language. Median accuracy scores varied with a maximum of 1 on a 5-point Likert-scare according to question's framing. Overall accuracy scores were 83–89 % for ‘nutrition in pregnancy’ versus 96–98 % for ‘red flags in pregnancy’. Inter-rater agreement was good to excellent for both topics.

**Conclusion:**

Although ChatGPT generated accurate recommendations regarding the tested indicators for nutrition and red flags during pregnancy, women should be aware of ChatGPT's limitations such as inconsistencies according to formulation, language and the woman's personal context.

**Innovation:**

Despite a growing interest in the potential use of artificial intelligence in healthcare, this is, to the best of our knowledge, the first study assessing potential limitations that may impact accuracy of ChatGPT-generated recommendations such as language and question-framing in key domains of perinatal health.

## Highlights

• ChatGPT may be of interest in perinatal health information.
• Interpretation of ChatGPT's answers is heterogeneous.
• The user's formulation and language influence ChatGPT's accuracy.

## 1 Introduction

Patient education is critical in promoting perinatal health and has been associated with positive effects both on maternal and neonatal outcomes [[1], [2], [3], [4]]. One of the keystones of patient education is the form in which information is delivered to the patient. Over the past decade, there has been growing interest in digital patient education materials which have proven their efficacy compared to traditional forms of patient information [[5], [6], [7], [8]]. More than three quarters of childbearing women appear to turn to the Internet for information about pregnancy related issues [9]. Nutrition and red flags during pregnancy, defined as life-threatening conditioning or potentially serious condition requiring extended attention, are two topics of specific interest in this context as a large body of research has revealed that the majority of pregnant women seek online information on nutrition (50–60 %) and red flags during pregnancy (30–50 %) at least once during pregnancy [[10], [11], [12], [13], [14], [15], [16]]. Accurate patient information on these two topics has been associated with positive dietary practices and uptake of appropriate care, thus reducing maternal and neonatal morbidity. However, patient information provided by health care workers is often inadequate due to limited time and resourcess [1,[17], [18], [19], [20], [21]].

The recent advancement of conversational artificial intelligence (AI) models, such as Chat Generative Pretrained Transformer (ChatGPT), opens up new possibilities in this context. ChatGPT is an AI-powered conversational model that utilizes deep learning techniques to generate human-like responses based on input queries. It may offer potential benefits in patient information compared to classic patient information such as easy and immediate availability [22]. Nevertheless, rigorous evaluation of the use of ChatGPT for perinatal patient information is currently non-existent [[23], [24], [25], [26], [27]].

In this study, we therefore aimed to assess the accuracy of ChatGPT-generated recommendations compared to existing national guidance with regard to nutritional recommendations and red flags in pregnancy. Secondary, we aimed to evaluate whether there is a difference in the accuracy of ChatGPT-generated information on the above-described topics depending on the applied wording of the question and according to the language in which we address this AI model.

## 2 Methods

We performed an accuracy study in which we compared the accuracy and reliability of recommendations generated by ChatGPT with existing guidance from professional organizations, which were considered as the key response.

### 2.1 Subtopics

The performance of ChatGPT was tested for two topics: (1) Nutrition in pregnancy and (2) Red flags in pregnancy. For each of these topics, we defined 10 subtopics for which we tested the accuracy of ChatGPT's response. Subtopics had to represent key aspects within these two topics of perinatal health information by two experts (HL + EM) and were validated by all authors.

For nutritional recommendations, the defined subtopics were safety and hazard of: wine, soft cheese, smoked salmon, seafood, charcuterie, and liver; necessity and dose of: folic acid supplementation, vitamin-D supplementation, iron supplementation, calcium supplementation.

For red flags during pregnancy, the following subtopics were defined: hazard of: abdominal trauma, absence of fetal movement in the first trimester, absence of fetal movement in the second/third trimester, vaginal bleeding in the third trimester, rupture of membranes in the third trimester, premature uterine contractions, and fever, suicidal thoughts, domestic violence, and food poisoning whatever term of pregnancy.

### 2.2 Key response elements

For each subtopic, existing guidelines stemming from professional societies were considered to develop the Key Responses. To facilitate the comparison with the content of the official guidelines, key elements were withdrawn from the identified recommendations by two of the experts (HL + EM) and validated by all authors. These key elements were considered as Key Response as their presence was mandatory in ChatGPT's response to properly inform patients.

As we aimed to challenge ChatGPT in English, French, German and Dutch, for each language, a different guideline was used, each stemming from a country where the assessed language is considered a national language (Table S1). For the red flags during pregnancy, no Key Responses were available in German (Switzerland, Germany or Austria), which is why this topic could only be tested in three languages.

The English and French key elements were formulated in the original language, as all raters are bilingual in French and English. The Dutch and German key elements were compiled by a Dutch and German native speaker (PdV and UW) who translated the key elements in English.

### 2.3 ChatGPT's answers and ranking

For ‘nutrition during pregnancy’ each subtopic was assessed by two different question framings. These were designed (by EM + HL) in order to test the impact of the formulation on the reliability of ChatGPT's response. These questions had to be compatible with questions a patient without any medical background would normally ask ChatGPT. The two different question framing were: *‘Is it dangerous to ….. during pregnancy.’* versus *‘Is it safe to …. during pregnancy.’* For the Dutch and German languages, these questions were translated by PdV and UW.

The French and English responses of ChatGPT were assessed and ranked by all raters in the original language. For the Dutch and German responses of ChatGPT, two native speakers (PdV and UW) translated ChatGPT's responses in English to allow all raters to properly compare ChatGPT's answers with the Key Response Elements.

To measure the accuracy of ChatGPT's generated answers, eight raters (AP, EG, EM, GF, HL, LP, PdV, UW) scored the data on a 5-point Likert scale as follows: Score of 1 (no accuracy): the answer can lead to harmfulness if followed literally by the patient. Score of 2: key elements are missing but the answer does not directly lead to harmfulness to the patient. Score of 3: The main key element is not explicitly mentioned but the answer is close to it. Score of 4: The main key elements are mentioned but not all. Score of 5: all key elements are correctly mentioned. The raters had different profiles and disciplines. All the raters used a new session with no previous conversation with the ChatGPT tool.

The study was performed using the AI-model version ChatGPT-4.

### 2.4 Statistical analysis

For all subtopics, the median accuracy score per topic and subtopic was calculated and given with its interquartile range.

The overall accuracy score for each topic was calculated by the sum of all the scores of the individual items in a topic and scaled in percentages of the maximum possible score for that topic. If an item was considered as ‘non-judgable’, it was not included in this calculation.

To determine the inter-rater reliability, we calculated the intra-class correlation coefficient (ICC) as a method to assess the agreement between the raters analyzing each subtopic. Raters were considered unique (all raters scored every subtopic) and a two-way mixed-effects model was chosen. ICCs were given per language separately for both topics with their 95 % confidence intervals (95 % CI). ICC results were interpreted according to the following criteria: poor (ICC < 0.50), moderate (0.50 ≤ ICC < 0.75), good (0.75 ≤ ICC < 0.90), and excellent (ICC ≥ 0.90) [28].

All statistical analyses were performed using Excel version 2016 and Stata® v13 software.

Ethical approval was not required since no patients were involved in the development of this research.

## 3 Results

All eight raters scored the accuracy of recommendations generated by ChatGPT compared to Key Response per subtopic for both topics. In Table 1, we present the median accuracy scores on a 5-point Likert scale per subtopicfor both subtopic, stratified per language.

**Table 1:** Median accuracy score per subtopic for accuracy of ChatGPT generated recommendations as compared to Key Response Elements, divided per language assessed.

Theme	Language								
	Dutch			English		French		German	
Nutrition during pregnancy	Median		IQR^c^	Median	IQR	Median	IQR	Median	IQR
*Indicator*									
*Safety and hazard of Question*^a^									
Wine	**A**	5	(5)	5	(5)	5	(5)	5	(5)
	**B**	5	(5)	5	(5)	5	(5)	5	(5)
Soft cheese	**A**	4	(4)	5	(5)	4	(4)	5	(5)
	**B**	5	(5)	5	(5)	5	(4, 5)	5	(5)
Smoked salmon	**A**	5	(5)	5	(5)	4	(4)	4	(4)
	B	5	(5)	5	(5)	4	(3, 4)	4	(4)
Sea food	A	5	(5)	5	(5)	5	(5)	5	(5)
	B	5	(5)	5	(5)	5	(5)	5	(5)
Charcuterie	A	4	(4)	5	(4, 5)	5	(5)	5	(5)
	B	4	(4, 5)	4	(4, 5)	5	(5)	5	(5)
Liver	A	5	(5)	5	(5)	5	(5)	5	(5)
	B	4	(4, 5)	4	(4, 5)	4	(4)	5	(5)

*Necessity and dose of Question*^b^									
Folic acid	A	4	(4)	4	(4)	4	(4)	4	(4)
	B	5	(5)	4	(4)	4	(4)	4	(4)
Vitamin D	A	5	(5)	5	(5)	5	(5)	5	(5)
	B	5	(5)	5	(5)	5	(5)	5	(5)
Iron	A	5	(5)	4	(3, 4)	5	(5)	5	(5)
	B	4	(4)	4	(3, 4)	5	(5)	5	(5)
Calcium	A	5	(4, 5)	5	(5)	5	(5)	5	(5)
	B	5	(5)	5	(5)	5	(4, 5)	5	(5)
Total theme score		**5**	**(4–5)**	**5**	**(4–5)**	**5**	**(4–5)**	**5**	**(4–5)**
Red Flags during pregnancy	Median		IQR	Median	Median	IQR	Median	IQR	IQR

*Indicator*									
*Hazard of*									
Abdominal trauma		5	(5)	5	(5)	5	(5)	Na^d^	Na
Absence fetal movement in the first trimester		5	(5)	5	(5)	5	(5)	Na	Na
Absence of fetal movement in the second/third trimester		3	(3, 4)	5	(5)	5	(5)	Na	Na
Vaginal bleeding third trimester		5	(5)	4	(4, 5)	5	(5)	Na	Na
Rupture of membranes third trimester		5	(5)	5	(5)	5	(5)	Na	Na
Premature uterine contractions		5	(5)	5	(5)	5	(5)	Na	Na
Fever whatever pregnancy term		3	(3, 4)	5	(5)	5	(5)	Na	Na
Suicidal thoughts whatever pregnancy term		5	(5)	5	(5)	5	(5)	Na	Na
Domestic violence whatever pregnancy term		5	(5)	5	(5)	5	(5)	Na	Na
Food poisoning whatever pregnancy term		Na	Na	5	(5)	5	(5)	Na	Na
*Total theme score*		**5**	**(5–5)**	**5**	**(5–5)**	**5**	**(5–5)**	**5**	**(5–5)**

Score were calculated on a 5-points Likert scale. 1 (no accuracy): the answer can lead to harmfulness if followed literally by the patient. Score of 2: key elements are missing but answer not directly leading to harmfulness to the patient. Score of 3: main key element is not explicitly mentioned but the answer is close to. Score of 4: main key elements are mentioned but not all. Score of 5: all key elements are correctly mentioned

a Question A refers to a positive question framing (‘safety’), question B to a negative question framing (‘hazard’).

b Question A refers to ‘necessity’, question B refers to ‘dose’.

c Inter quartile range.

d Not attributable.

**Fig. 1:**
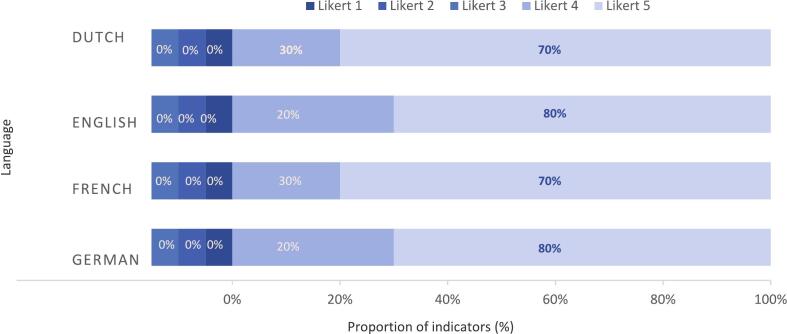
Proportion of median accuracy score for each possible Likert score (5-points scale) of ChatGPT's answer as compared to Key Response per language for ‘Nutrition in pregnancy’.

**Fig. 2:**
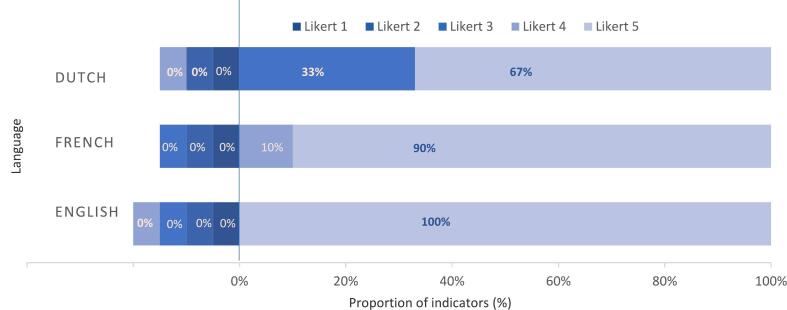
Proportion of median accuracy scores for each possible Likert score (5-points scale of ChatGPT's answer as compared to Key Response per language for ‘Red flags in pregnancy’. (For interpretation of the references to color in this figure legend, the reader is referred to the web version of this article.)

For ‘Nutrition during pregnancy’, the median accuracy score was 5.0 (IQR 4–5) on a 5-point Likert scale independently of the language, indicating ChatGPT's responses consistently included all key elements from the Key Responses. In Dutch and French, 30 % of the subtopics were classified with a median accuracy score of 4 versus 70 % with a 5. In English and German, 20 % of the subtopics were scaled with a median accuracy score of 4 versus 80 % with a median accuracy score of 5. For ‘Red flags in pregnancy’, the median accuracy score was 5 (IQR 5–5) for all languages. ChatGPT's answers in French and English obtained a median accuracy score of 5 in 100 % and 90 % of subtopics, respectively. The Dutch responses were rated with a median accuracy score of 4 in 33 % of subtopics and with a score of 5 in 67 % of subtopic. Proportions of median accuracy scores are presented in Fig. 1, Fig. 2. ChatGPT recommended to contact a health care provider for addition nutritional recommendations in 9/10 subtopics in English and French, in 6/10 subtopics in Dutch and in 0/10 subtopics in German. For red flags in pregnancy, ChatGPT recommended to contact a health care provider in all subtopics independently of the language. (Supporting information).

When testing the accuracy of ChatGPT according to formulation, we observed variations in response content depending on how the questions were framed. In French and English, 2/10 subtopics had a different median accuracy score according to question framing versus 4/10 subtopics in Dutch and, these differences reached a maximum of 1 point on the Likert scale. Although the answers provided in box 1 lacked key elements, they illustrate ChatGPT's variability according to the formulation of the user's question.

Box 1 **Users framing questions negatively to ChatGPT:**

Is it dangerous to eat smoked salmon during pregnancy?

**ChatGPT's answer:**

Smoked salmon is a healthy food and a good source of omega-3 fatty acids. However, there are some things to consider when it comes to consuming smoked salmon during pregnancy. It's important to only eat smoked salmon from reliable sources and to pay attention to the quality and freshness of the product. Some varieties of smoked salmon can be contaminated with Listeria or other harmful bacteria, which can increase the risk of food poisoning during pregnancy.

Additionally, smoked salmon can also have a higher salt content than other types of fish. Excessive salt intake can lead to high blood pressure, which poses an increased risk of complications such as preeclampsia during pregnancy.

For these reasons, it's recommended to limit the consumption of smoked salmon during pregnancy. If you do want to eat smoked salmon, make sure it comes from a reliable source and is consumed in moderation.

In summary, smoked salmon can be safely consumed in moderation during pregnancy if it comes from a reliable source and attention is paid to quality, freshness, and salt content. However, it's best to limit consumption to minimize the risk of food poisoning and complications during pregnancy.

**Users framing questions positively to ChatGPT:**

Is it safe to eat smoked salmon during pregnancy?

**ChatGPT's answer:**

It is recommended that pregnant women exercise caution when consuming salmon. While smoked salmon is certainly delicious, it may be contaminated with Listeria or other harmful bacteria that can be dangerous during pregnancy.

For this reason, it is recommended that pregnant women only consume salmon that is well-cooked and not raw or undercooked. This will kill most of the bacteria that may be present in the salmon. It is also important to pay attention to the source of the salmon and ensure that it comes from a reliable source.

Overall, pregnant women should always consult with their doctor before making any changes to their diet or consuming certain foods. The doctor can provide individual recommendations based on the woman's health and medical background.

**Key Response in the United Kingdom**

Smoked fish has been associated to an increased risk of Listeria. Therefore, people at higher risk of serious infection (including people who are pregnant) should only eat smoked fish products that have been thoroughly cooked. When cooking smoked fish products at home, make sure they are steaming hot all the way through.

Alt-text: Box 1

**Fig. 3:**
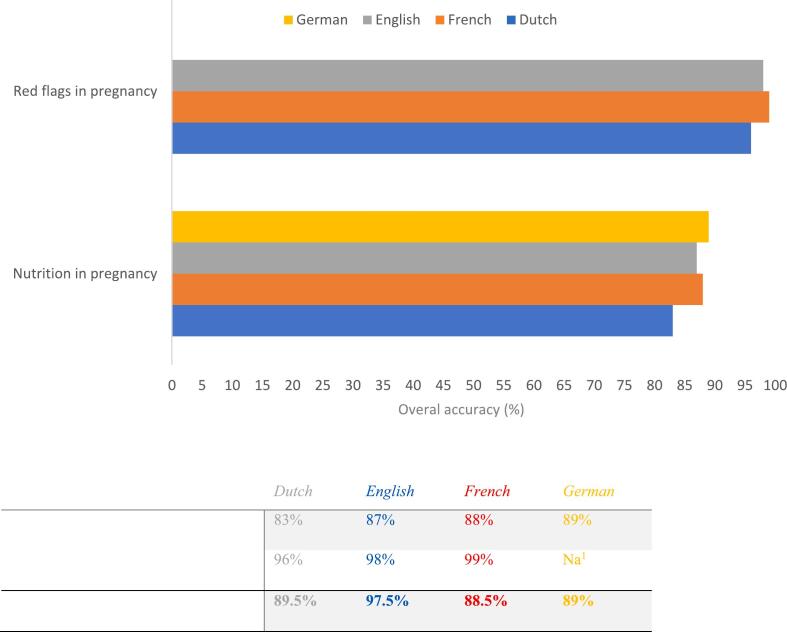
Overall accuracy of ChatGPT in perinatal health information per topic and per language compared to key response.

The overall accuracy scores for ‘Nutrition in pregnancy’ varied between 83 and 89 % depending on the language and 96–98 % for ‘Red flags in pregnancy’ (Fig. 3).

Although ChatGPT's answers for the Dutch subtopics scoring 3 on the Likert scale were scientifically correct according to the experts, they were not entirely conforming to the Key Response based on the Dutch guideline. An example is provided in box 2.

Box 2 **Question formulated to ChatGPT:**

I am in the third trimester of my pregnancy and I have lost some amniotic fluid. Should I go to the maternity hospital?

**ChatGPT's answer:**

Yes, if you have lost some amniotic fluid during your third trimester of pregnancy, you should go to the maternity hospital immediately. Loss of amniotic fluid could be a sign that your water has broken prematurely, which can increase the risk of infection and other complications for you and your baby. It is important to seek medical attention promptly so that healthcare professionals can assess your condition and take appropriate action. Depending on the amount of fluid loss and your overall health, your healthcare provider may recommend inducing labor or taking other measures to protect your health and that of your baby. If you experience any other symptoms such as vaginal bleeding, contractions, or fever, or if you feel that something is not right, seek medical help immediately.

**Key Response in the Netherlands:**

Rupture of the membranes may announce the beginning of labor. After 37 weeks, this may announce labor and it is important to check the color of the liquid (clear, or yellowish / green). Sometimes, liquid can be a little bit pink due to the fact that amniotic fluid can be mixed with a little bit of blood which is normal when the cervix starts dilating. Try to keep some of the liquid to show it to your midwife so she can check the color when she comes to your house for an obstetrical check-up. If contractions do not start after 24 h after rupturing the membranes, your midwife will send you for a medical check-up to the hospital.

This example brings to light that ChatGPT did not take into account the national context of the Netherlands where, instead of going to the hospital, low-risk women will call their midwife to perform medical check-up at home. Women will only be sent to the hospital if there are signs of infection or no contractions after 24 h of expective management. Although the experts considered that ChatGPT's response did not cause any harm to the fetus nor the mother, it may cause confusion among women in the Netherlands when applied literally. Thus, it was ranked lower than in other languages. When adding the country of residence to the question, the content of ChatGPT's answer did not change.

Alt-text: Box 2

The experts noticed that ChatGPT's answers for many subtopics were more complete than existing guidelines, with more nuances and easier interpretable recommendations as compared to the Key Response.

The agreement between raters is presented in Table 2. For ‘Nutrition in pregnancy’, the ICC varied from 0.78 (95 %CI 0.77–0.82) to 0.82 (95 %CI 0.79–0.84) depending on the language. This inter-rater reliability can be considered as ‘good’ for all four languages. The ICC for ‘Red Flags in pregnancy’ varied from 0.83 (95 %CI 0.79–0.89) to 0.95 (95 % CI 0.93–1.0). Inter-rater variability was considered as ‘good’ for the Dutch answers and ‘excellent’ for the French and English answers (Table 2).

**Table 2:** Inter rater variability of the accuracy of ChatGPT, per topic and per language, in perinatal health information for patients calculated by intra-class correlation.

	Dutch ICC	95 %CI	French ICC	95 %CI	English ICC^a^	95 % CI^b^	German ICC	95 %CI
Theme								
Nutrition during pregnancy	0.81	0.78–0.86	0,79	0.74–0.83	0.82	0.79–0.84	0.78	0.77–0.82
Red flags during pregnancy	0.83	0.79–0.89	0.87	0.82–0.91	0.95	0.93–1.0	–	

ICC can be considered poor (ICC < 0.50), moderate (0.50 < ICC < 0.75), good (0.75 < ICC < 0.90), and excellent (ICC > 0.90).

a intra class correlation coefficient.

b confidence interval.

## 4 Discussion and conclusion

### 4.1 Discussion

In this study, we explored the accuracy of patient information provided by ChatGPT on nutrition and the identification of red flags during pregnancy.

The accuracy of ChatGPT for the subtopics tested in this study appeared excellent. Although agreement between the raters was not 100 %, inter-rater reliability was good to excellent. The experts noticed that ChatGPT's answers were often even more comprehensive than existing guidelines. These differences may be explained by the fact that clinical practice guidelines perform a quality assessment of the included data whereas ChatGPT does not function with such a grading system resulting in the integration of different sources. ChatGPT was trained on documentation available in 2021 illustrating ChatGPT's limitations in providing the most up-to-date recommendations. Furthermore, ChatGPT includes in its training documents that may be contradictory, such as non-scientific sources including pseudo-scientific or vernacular health statements.

Although our findings may be promising, our study highlights several deficiencies in using ChatGPT for patient information. Indeed, we report variations in the median accuracy score depending on the language used. Although none of ChatGPT's answers resulted in direct harm to the fetus or the mother, ChatGPT may not allways be consistent in recommending to consult with a health care provider according to the applied language, which may falsely reassure patients. This finding is in line with some other studies, reporting a decrease in the performance of ChatGPT in languages other than English according to the complexity of the question. This could be explained by the fact that most of the input data of ChatGPT are written in English. By translating this information, certain nuances due to language specific expressions as well as national context may get lost, reducing ChatGPT's accuracy [[29], [30], [31], [32]]. Also, we report that ChatGPT does not seem to adapt its answers to the local healthcare context of the patient when this is not detailed any further in the question, resulting in conflicting recommendations compared to national guidelines [26,33]. In addition, ChatGPT bases its interactions on a vast amount of data which has been shown to underrepresent minority groups [34]. This could further increase ChatGPT's inability to consider women from these groups. In this context, it would also be interesting to investigate comprehensiveness of ChatGPT's responses among the pregnant population. Although the raters considered that ChatGPT generated comprehensible recommendations, the assessment of the use of plain language requires specific competencies and based on the evaluation of the raters we cannot simply assume that comprehensiveness would at the same level among the pregnant population. As such, this could also contribute to increased health inequalities, emphasizing the need to perform further surveys among pregnant women and their comprehension of ChatGPT provided recommendations. Our results indicate variations in ChatGPT's accuracy based on the way questions are framed. This finding is relevant as it may reduce the reliability of ChatGPT. ChatGPT's reliability is also affected by its contextual behaviour which was confirmed by the fact that ChatGPT sometimes altered its answers depending on the previous question. The same is true for the way in which questions are framed. Several studies have highlighted the impact of positive prompting questions which appear to encourage ChatGPT to generate a different type of response as compared to negative prompting questions, reducing ChatGPT's realiability [[35], [36], [37], [38]].

Although we did not find any data on the proportion of women that currently turns to ChatGPT for information on pregnancy related issues, the AI tool can be considered as a promising tool compared to classical methods of patient information as it is accessible and can provide information in patients' preferred language. This is of great relevance for the topics tested in this study which often require immediate attention and for which interpretation services are not always readily available [39,40]. Alternatives such as patient leaflets illustrated by pictograms are limited to a selection of abnormal conditions in pregnancy and not available in all languages compared to the information generated by ChatGPT.

Our study had several strengths. The accuracy of ChatGPT was assessed by a Likert Scale, allowing the expression of a certain degree of accuracy. As the raters assessed accuracy independently, the risk of cognitive bias was considered minimal. The different disciplines and profiles of the raters provided an added value by showing that the reliability of responses is not a priori correlated to the raters' background. The testing of the AI-model in different languages can be considered as another important strength in our study, as restricting studies of this type to English only may limit the understanding of ChatGPT's accuracy. Some limitations should also be considered. Firstly, we tested only two topics of perinatal health information, thus, our findings cannot be extrapolated to other topics of perinatal health information. Secondly, although we tried to construct our Key Response Elements by considering several guidelines from countries where the respective languages are official, our approach may not have covered every possible source. Thirdly, improving accuracy and reliability are never-ending processes, as ChatGPT is constantly evolving. Fourth, we did not test whether ChatGPT is able to adapt its answers to the patient's local context by giving more specifications other than the language and the country of residence. Finally, some recent developments intend to combine conversational agents with search engine results. The adequacy of such combination deserves further evaluation when they will be as widely available as ChatGPT is.

### 4.2 Innovation

There has been a growing interest in the potential use of artificial intelligence in healthcare. However, accuracy of ChatGPT as an information source for women to educate themselves on topics related to perinatal health has only been poorly investigated despite the fact that women of procreative age will easily seek answers on pregnancy related issues by using artificial intelligence tools such as ChatGPT. To the best of our knowledge, performance of ChatGPT has not yet been investigated when it is confronted to factual questions regarding key topics in perinatal health. To assess potential limitations of ChatGPT, we investigate its accuracy according to different languages and to the wording of the question as these potential confounders have currently not yet been explored.

### 4.3 Conclusion

While ChatGPT may appear to be a valuable tool for improving perinatal health information by delivering seemingly accurate content, it is essential that patients are made aware of its limitations. Indeed, our study has identified certain drawbacks including inconsistent accuracy depending on the user's formulation and language, as well as its inability to take into account the patient's personal context.

## Detail of ethics approval

Not applicable.

## Funding

The research activities of Michael Ceulemans are funded by the Internal Funds KU Leuven (PDMt1/23/020).

## CRediT authorship contribution statement

**P.L.M. de Vries:** Writing – original draft, Visualization, Validation, Methodology, Investigation, Formal analysis, Data curation, Conceptualization. **D. Baud:** Writing – review & editing, Supervision, Methodology. **S. Baggio:** Writing – review & editing, Visualization, Methodology, Formal analysis. **M. Ceulemans:** Writing – review & editing. **G. Favre:** Writing – review & editing, Validation, Methodology, Investigation. **E. Gerbier:** Writing – review & editing, Validation, Methodology, Investigation. **H. Legardeur:** Writing – review & editing, Validation, Methodology, Investigation. **E. Maisonneuve:** Writing – review & editing, Validation, Methodology, Investigation. **C. Pena-Reyes:** Writing – review & editing. **L. Pomar:** Writing – review & editing, Validation, Methodology, Investigation. **U. Winterfeld:** Writing – review & editing, Validation, Methodology, Investigation. **A. Panchaud:** Writing – review & editing, Validation, Supervision, Methodology, Investigation, Conceptualization.

## Declaration of competing interest

The authors declare that they have no known competing financial interests or personal relationships that could have appeared to influence the work reported in this paper.

## Appendix A Supplementary data

Supplementery material 1: Availability of data per topic and per language Supplementary material 1

Supplementary material 2 Tabls S1: Sources for key response elements per topic and per language Supplementary material 2

## References

[1] Girard A.W., Olude O. (2012). Nutrition education and counselling provided during pregnancy: effects on maternal, neonatal and child health outcomes. Paediatr Perinat Epidemiol.

[2] Hong K., Hwang H., Han H. (2021). Perspectives on antenatal education associated with pregnancy outcomes: systematic review and meta-analysis. Women Birth.

[3] Jain J., Moroz L. (2017). Strategies to reduce disparities in maternal morbidity and mortality: patient and provider education. Semin Perinatol.

[4] Ellington K. (2018). Web-based perinatal education for the new obstetrical patient: a quality improvement project. J Perinat Educ.

[5] Lupton D., Pedersen S. (2016). An Australian survey of women’s use of pregnancy and parenting apps. Women Birth.

[6] Skar J.B., Garnweidner-Holme L.M., Lukasse M., Terragni L. (2018). Women’s experiences with using a smartphone app (the pregnant+ app) to manage gestational diabetes mellitus in a randomised controlled trial. Midwifery.

[7] Parsa S., Khajouei R., Baneshi M.R., Aali B.S. (2019). Improving the knowledge of pregnant women using a pre-eclampsia app: a controlled before and after study. Int J Med Inform.

[8] Ledford C.J.W., Canzona M.R., Cafferty L.A., Hodge J.A. (2016). Mobile application as a prenatal education and engagement tool: a randomized controlled pilot. Patient Educ Couns.

[9] Declercq E.R., Sakala C., Corry M.P., Applebaum S. (2007). Listening to mothers II: report of the second National U.S. survey of Women’s childbearing experiences: conducted January-February 2006 for childbirth connection by Harris interactive(R) in partnership with Lamaze international. J Perinat Educ.

[10] Lu Y., Zhang Z., Min K., Luo X., He Z. (2021). Divers Divergence Dialogue 16th Int Conf IConference 2021 Beijing China March 17-31 2021 Proc IConference Conf 16th 2021 Online.

[11] Mackintosh N., Agarwal S., Adcock K. (2020). Online resources and apps to aid self-diagnosis and help seeking in the perinatal period: a descriptive survey of women’s experiences. Midwifery.

[12] Sayakhot P., Carolan-Olah M. (2016). Internet use by pregnant women seeking pregnancy-related information: a systematic review. BMC Pregn Childbirth.

[13] Bjelke M., Martinsson A.K., Lendahls L., Oscarsson M. (2016). Using the internet as a source of information during pregnancy - a descriptive cross-sectional study in Sweden. Midwifery.

[14] Gao L., ling, Larsson M, Luo S yuan. (2013). Internet use by Chinese women seeking pregnancy-related information. Midwifery.

[15] Jacobs E.J.A., van Steijn M.E., van Pampus M.G. (2019). Internet usage of women attempting pregnancy and pregnant women in the Netherlands. Sex Reprod Healthc.

[16] Hämeen-Anttila K., Nordeng H., Kokki E. (2014). Multiple information sources and consequences of conflicting information about medicine use during pregnancy: a multinational internet-based survey. J Med Internet Res.

[17] Goodarzi-Khoigani M., Baghiani Moghadam M.H., Nadjarzadeh A., Mardanian F., Fallahzadeh H., Mazloomy-Mahmoodabad S. (2018). Impact of nutrition education in improving dietary pattern during pregnancy based on Pender’s health promotion model: a randomized clinical trial. Iran J Nurs Midwifery Res.

[18] Lee A., Newton M., Radcliffe J., Belski R. (2018). Pregnancy nutrition knowledge and experiences of pregnant women and antenatal care clinicians: a mixed methods approach. Women Birth J Aust Coll Midwives.

[19] Lucas C., Charlton K.E., Yeatman H. (2014). Nutrition advice during pregnancy: do women receive it and can health professionals provide it?. Matern Child Health J.

[20] Mwilike B., Nalwadda G., Kagawa M., Malima K., Mselle L., Horiuchi S. (2018). Knowledge of danger signs during pregnancy and subsequent healthcare seeking actions among women in urban Tanzania: a cross-sectional study. BMC Pregn Childbirth.

[21] Yunitasari E., Matos F., Zulkarnain H. (2023). Pregnant woman awareness of obstetric danger signs in developing country: systematic review. BMC Pregn Childbirth.

[22] Johnson D., Goodman R., Patrinely J. (2023). Assessing the accuracy and reliability of AI-generated medical responses: an evaluation of the Chat-GPT Model. Res Sq.

[23] Kung T.H., Cheatham M., Medenilla A. (2023). Performance of ChatGPT on USMLE: potential for AI-assisted medical education using large language models. PLOS Digit Health.

[24] Miller D.D., Brown E.W. (2018). Artificial intelligence in medical practice: the question to the answer?. Am J Med.

[25] Javaid M., Haleem A., Singh R.P. (2023). ChatGPT for healthcare services: an emerging stage for an innovative perspective. BenchCouncil Trans Benchmarks Stand Eval.

[26] Sallam M. (2023). ChatGPT utility in healthcare education, research, and practice: systematic review on the promising perspectives and valid concerns. Healthcare.

[27] Khan R.A., Jawaid M., Khan A.R., Sajjad M. (2023). ChatGPT - reshaping medical education and clinical management. Pak J Med Sci.

[28] Koo T.K., Li M.Y. (2016). A guideline of selecting and reporting Intraclass correlation coefficients for reliability research. J Chiropr Med.

[29] Shao C.Y., Li H., Liu X.L. (2023). Appropriateness and comprehensiveness of using ChatGPT for perioperative patient education in thoracic surgery in different language contexts: survey study. Interact J Med Res.

[30] Ando K., Sato M., Wakatsuki S. (2024). A comparative study of English and Japanese ChatGPT responses to anaesthesia-related medical questions. BJA Open.

[31] Fang C., Wu Y., Fu W. (2023). How does ChatGPT-4 preform on non-English national medical licensing examination? An evaluation in Chinese language. PLOS Digit Health.

[32] Lai Viet Dac, Ngo Nghia Trung, Veyseh Amir Pouran Ben (2023). ChatGPT Beyond English: Towards a Comprehensive Evaluation of Large Language Models in Multilingual Learning. https://arxiv.org/abs/2304.05613.

[33] Sharma S., Pajai S., Prasad R. (2023). A critical review of ChatGPT as a potential substitute for diabetes educators. Cureus.

[34] Panch T., Pearson-Stuttard J., Greaves F., Atun R. (2019). Artificial intelligence: opportunities and risks for public health. Lancet Digit Health.

[35] Farooqui M, Siddiquei M, Kathpal S. Framing assessment questions in the age of artificial intelligence: evidence from ChatGPT 3.5. Emerg Sci J.

[36] Bhattacharya M., Pal S., Chatterjee S. (2024). ChatGPT’s scorecard after the performance in a series of tests conducted at the multi-country level: a pattern of responses of generative artificial intelligence or large language models. Curr Res Biotechnol.

[37] Dave T., Athaluri S.A., Singh S. (2023). ChatGPT in medicine: an overview of its applications, advantages, limitations, future prospects, and ethical considerations. Front Artif Intell.

[38] Gianola S., Bargeri S., Castellini G. (2024). Performance of ChatGPT compared to clinical practice guidelines in making informed decisions for lumbosacral radicular pain: a cross-sectional study. J Orthop Sports Phys Ther.

[39] Eslier M., Deneux-Tharaux C., Schmitz T. (2023). Association between language barrier and inadequate prenatal care utilization among migrant women in the PreCARE prospective cohort study. Eur J Pub Health.

[40] Sentell T., Chang A., Ahn H.J., Miyamura J. (2016). Maternal language and adverse birth outcomes in a statewide analysis. Women Health.

