# Supplementary material for: Enhancing perinatal health patient information through ChatGPT – An accuracy study
Source: PEC Innov. 2025 Feb 10;6:100381. doi: 10.1016/j.pecinn.2025.100381 (PMC11872132; doi:10.1016/j.pecinn.2025.100381)
Supplement: Supplementery material 1: Availability of data per topic and per language [file mmc1.docx]

***PATIENT EDUCATION AND COUNSELING***

**CHECKLIST for the preparation of papers**

Please **ensure** that your paper conforms to the following guidelines:

**SPELLING**

☒ In the manuscript either US or UK usage should be followed but not a mixture of these.

**AFFILIATIONS**

☒Forenames for all authors in the author list; no titles like Prof., Dr., etc.

☒Affiliations must appear in English

NA In the affiliations use USA and UK instead of United States and United Kingdom

☒ The following should be followed for all authors’ affiliations: only Department,

University, City and Country

☒Provide full correspondence details (address including country, telephone, fax and e-mail address) for the corresponding author listed separately using the wording “Corresponding author at”

**ABSTRACT**

☒ Abstracts should be structured and have the following headings:

Objectives; Methods; Results; Conclusions; Practice implications

**MAIN TEXT**

☒All Original Articles must contain a first order heading section “Discussion and

Conclusion” and three second order headings: (1) Discussion, (2) Conclusion, (3) Practice implications. Sub-headings are only permitted within the Discussion.

**DECLARATION OF COMPETING INTERESTS**

☒All articles must include a separate file containing a statement declaring any competing interests that relate to any authors. The below is the format. Please click either of the two options and submit the form.

☒The authors declare that they have no known competing financial interests or personal relationships that could have appeared to influence the work reported in this paper.

☐The authors declare the following financial interests/personal relationships which may be considered as potential competing interests:

**REFERENCES**

☒Abbreviations for the names of journals in the reference list should follow Index

Medicus (e.g. JAMA should read J Amer Med Assoc, BMJ should read Brit Med J). The abbreviation for Patient Education and Counseling is Patient Educ Couns

☒Issue numbers and months must not be included in the reference list (only year, volume numbers and page range are required)

☒Page ranges in the reference list should appear as follows, e.g. 310-5

☒ Titles of non English publications should be given in English language, between [ ]

**For further details see the extended guidelines**
